# Supplementary figures and images for: Obesity Modulates the Gut Microbiome in Triple-Negative Breast Cancer
Source: Nutrients. 2021 Oct 19;13(10):3656. doi: 10.3390/nu13103656 (PMC8539565; doi:10.3390/nu13103656)

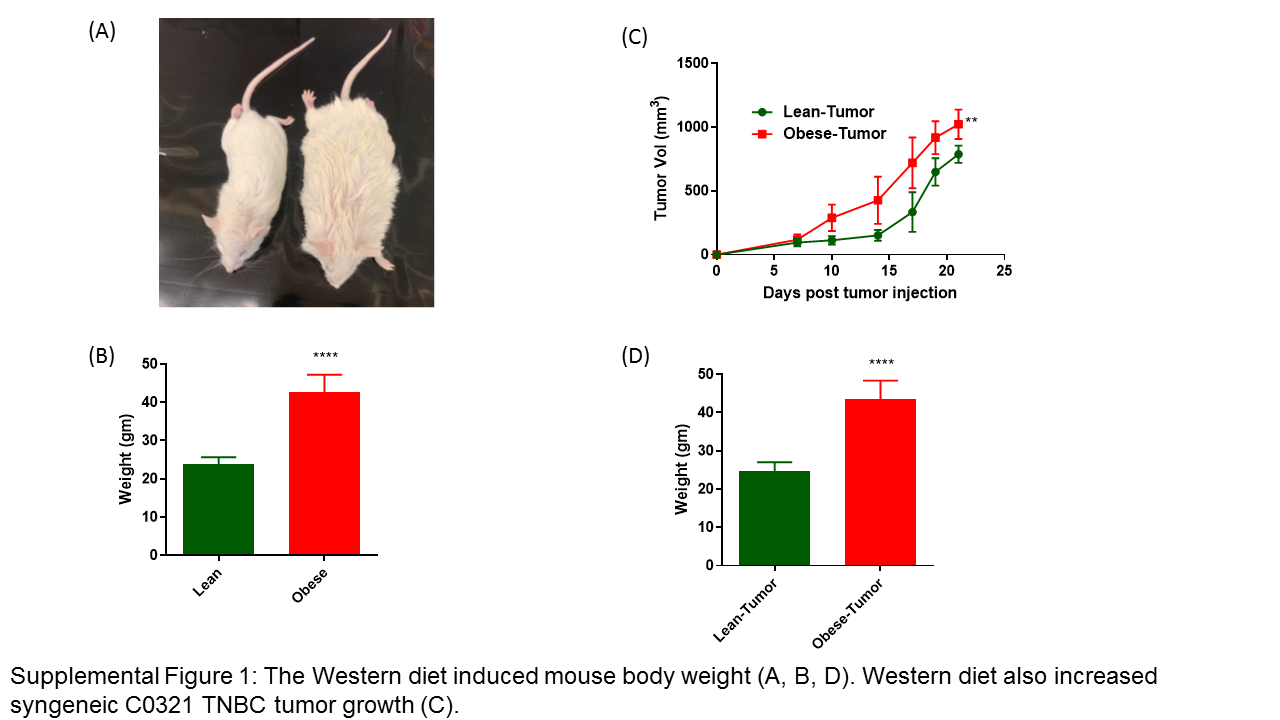

Supplement: Supplementary file 1 [file nutrients-13-03656-s001.zip › Supplemental materials/Slide1.TIF]

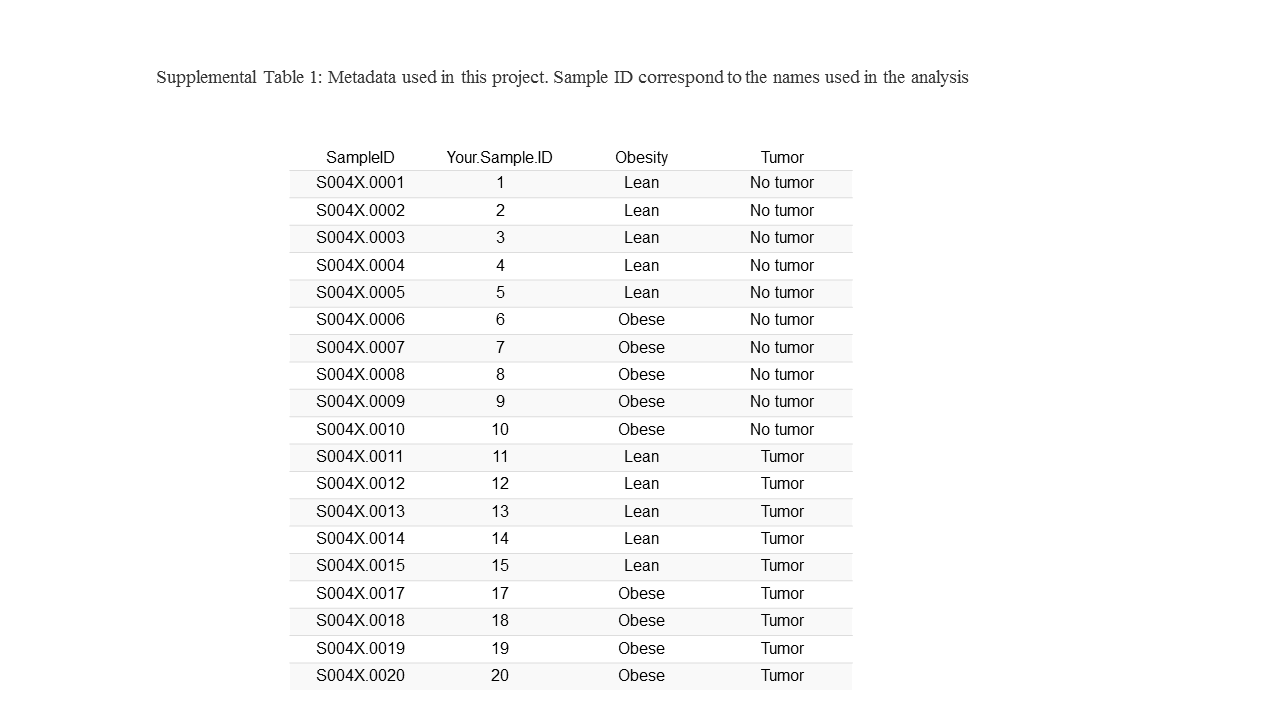

Supplement: Supplementary file 1 [file nutrients-13-03656-s001.zip › Supplemental materials/Slide2.TIF]

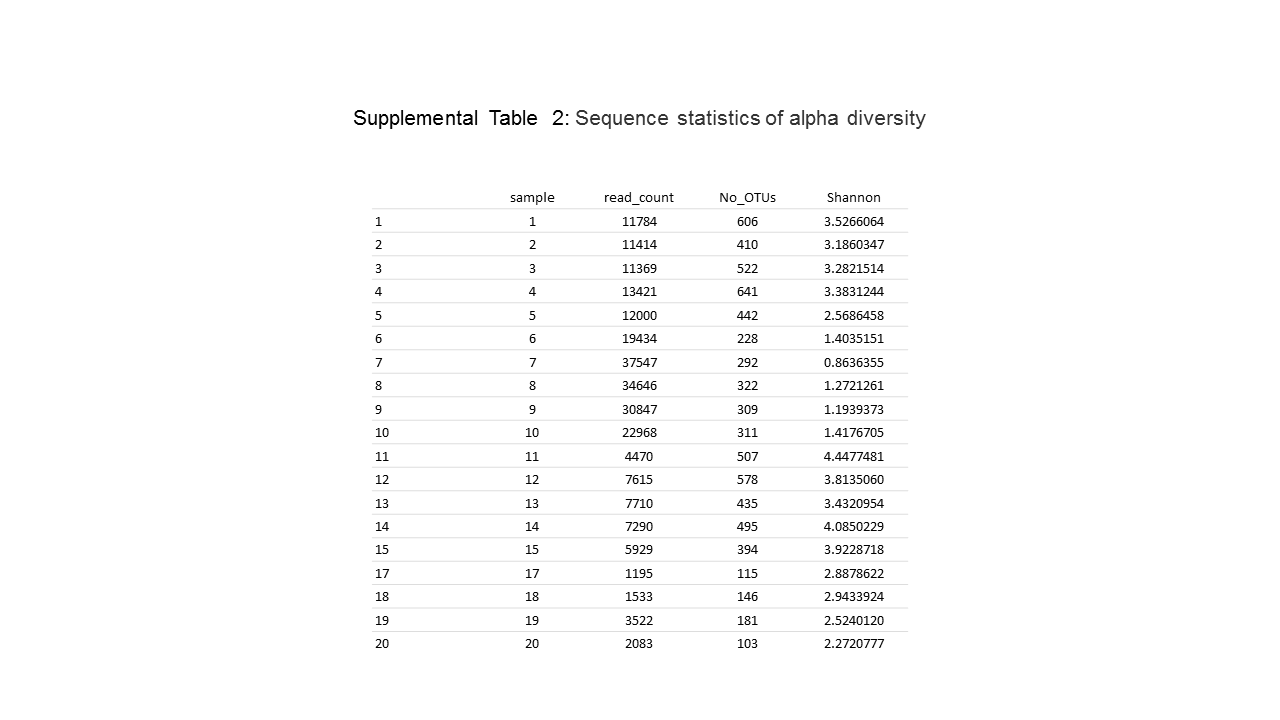

Supplement: Supplementary file 1 [file nutrients-13-03656-s001.zip › Supplemental materials/Slide3.TIF]

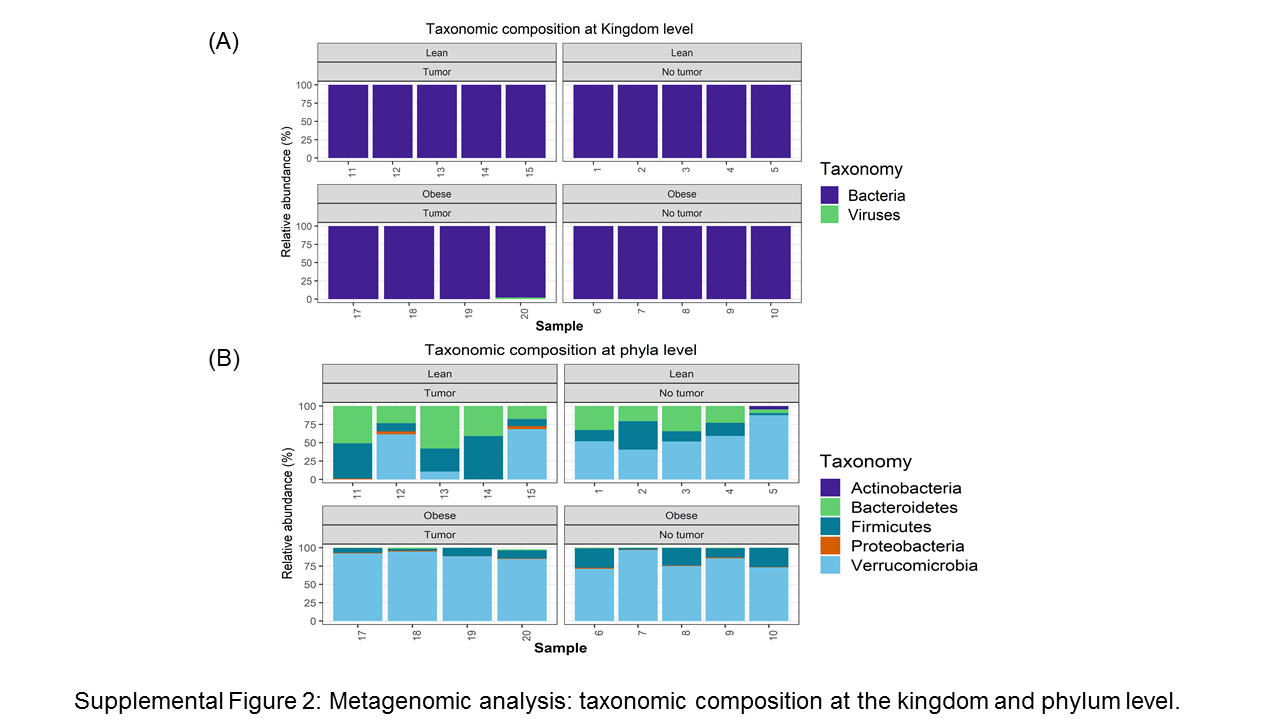

Supplement: Supplementary file 1 [file nutrients-13-03656-s001.zip › Supplemental materials/Slide4.TIF]

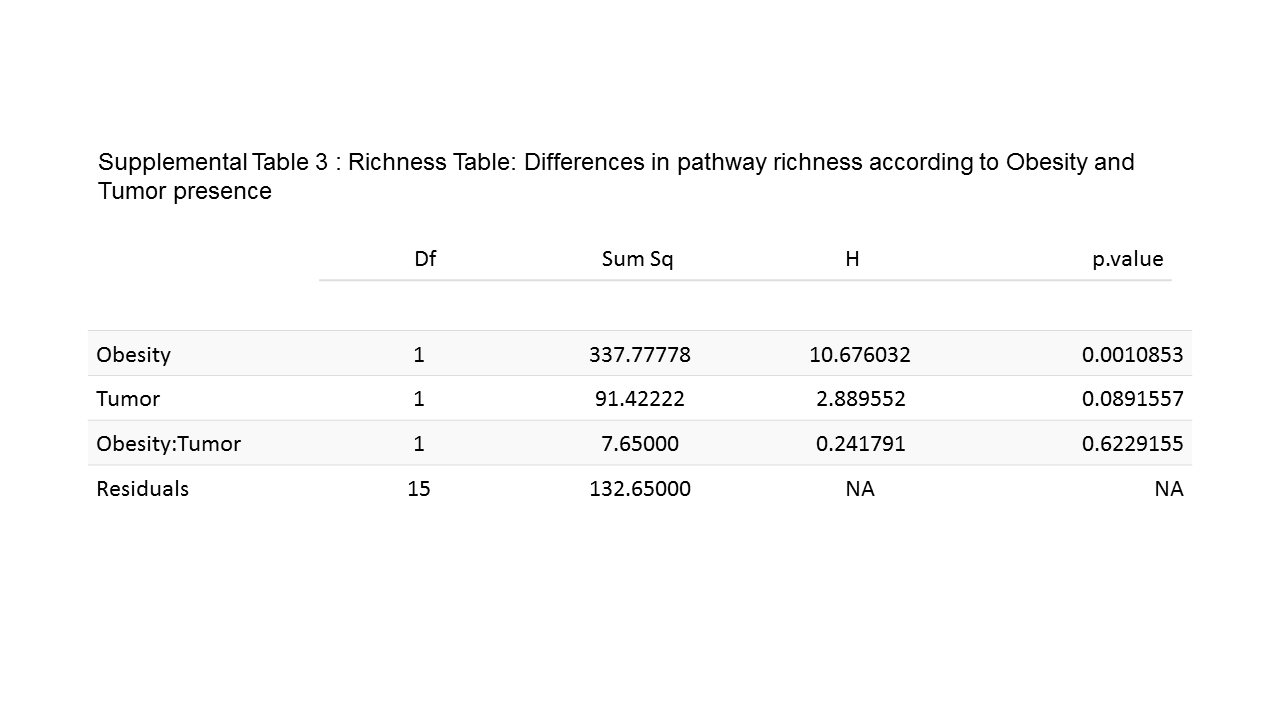

Supplement: Supplementary file 1 [file nutrients-13-03656-s001.zip › Supplemental materials/Slide5.TIF]
